# Tumor Immunotargeting Using Innovative Radionuclides

**DOI:** 10.3390/ijms16023932

**Published:** 2015-02-11

**Authors:** Françoise Kraeber-Bodéré, Caroline Rousseau, Caroline Bodet-Milin, Cédric Mathieu, François Guérard, Eric Frampas, Thomas Carlier, Nicolas Chouin, Ferid Haddad, Jean-François Chatal, Alain Faivre-Chauvet, Michel Chérel, Jacques Barbet

**Affiliations:** 1 Nantes-Angers Cancer Research Center (CRCNA), University of Nantes, Inserm UMR 892, CNRS UMR 6299, Nantes 44007, France; E-Mails: caroline.rousseau@ico.unicancer.fr (C.R.); caroline.milin@chu-nantes.fr (C.B.-M.); francois.guerard@univ-nantes.fr (F.G.); eric.frampas@chu-nantes.fr (E.F.); thomas.carlier@chu-nantes.fr (T.C.); alain.faivre-chauvet@univ-nantes.fr (A.F.-C.); michel.cherel@univ-nantes.fr (M.C.); jacques.barbet@univ-nantes.fr (J.B.); 2 Department of Nuclear Medicine, University Hospital, Nantes 44093, France; E-Mail: cedric.mathieu@chu-nantes.fr; 3 Department of Nuclear Medicine, Institut de Cancérologie de l’Ouest (ICO)-Site Gauducheau, Saint-Herblain 44805, France; 4 Department of Radiology, University Hospital, Nantes 44093, France; 5 AMAROC, ONIRIS, Nantes 44300, France; E-Mail: nicolas.chouin@oniris-nantes.fr; 6 Physics Department, Groupement d’Intérêt Public Arronax, Nantes 44817, France; E-Mails: ferid.haddad@univ-nantes.fr (F.H.); chatal@arronax-nantes.fr (J.-F.C.)

**Keywords:** innovative radionuclides, alpha particle-emitting radionuclides, alpha-immunotherapy, radioimmunotherapy, immuno-PET

## Abstract

This paper reviews some aspects and recent developments in the use of antibodies to target radionuclides for tumor imaging and therapy. While radiolabeled antibodies have been considered for many years in this context, only a few have reached the level of routine clinical use. However, alternative radionuclides, with more appropriate physical properties, such as lutetium-177 or copper-67, as well as alpha-emitting radionuclides, including astatine-211, bismuth-213, actinium-225, and others are currently reviving hopes in cancer treatments, both in hematological diseases and solid tumors. At the same time, PET imaging, with short-lived radionuclides, such as gallium-68, fluorine-18 or copper-64, or long half-life ones, particularly iodine-124 and zirconium-89 now offers new perspectives in immuno-specific phenotype tumor imaging. New antibody analogues and pretargeting strategies have also considerably improved the performances of tumor immunotargeting and completely renewed the interest in these approaches for imaging and therapy by providing theranostics, companion diagnostics and news tools to make personalized medicine a reality.

## 1. Introduction

Radiolabeled antibodies have been developed for imaging and therapy purposes in oncology since more than 30 years ago. The use of anti-carcinoembryonic antigen (CEA) radiolabeled polyclonal antibodies for the detection of cancers by “external photoscanning” was reported in 1978 by David Goldenberg and collaborators [1]. However, the real starting point was the discovery of the hybridoma technology [2], which quickly offered a series of new reagents specifically recognizing tumor cells [3,4]. One of the first applications envisaged for these new monoclonal antibodies (mAbs) was tumor imaging by immunoscintigraphy [4]. Iodine-131, used in clinical practice for imaging and treatment of differentiated thyroid carcinoma, was used to label mAbs, despite a relatively high-energy gamma emission. However, immunoscintigraphy developments met several problems: intact immunoglobulin, with long half-life, needed several days to provide good contrast images and some radio-labeled mAbs did not allow tumor detection due to poor retention of radioactivity in tumors. The first problem triggered intense research on the use of mAb fragments and later-on on pretargeting approaches. The second prompted to the use of radioactive metals, such as indium-111, which remain trapped in tumor cells after mAb internalization, contrary to iodine, which is rapidly excreted in the form of iodo-tyrosine after degradation of the internalized radio-labeled mAb [5]. Finally when the quality of images started to improve, in part with the introduction of pretargeting approaches [6], positron emission tomography (PET) had developed showing impressive results in solid tumor and lymphoma imaging, despite the use of the non-specific tracer [^18^F]-fluoro deoxy glucose (FDG).

In the meantime, mAb immunogenicity had been reduced by the introduction of chimeric, humanized or human antibodies [7] and targeted therapies using high amounts of naked mAbs experienced an important development in hematology with the anti-CD20 rituximab in B non-Hodgkin lymphoma (NHL) and oncology with the anti-HER2 trastuzumab in breast carcinoma (BC) [8,9]. Based on the old concept of “magic bullets”, mAbs carrying radionuclides are now showing their potential in cancer treatment, by radioimmunotherapy (RIT) in NHL and several solid tumors [10,11,12]. Now, because of access to innovative PET emitters, mAbs are also considered for imaging purpose, immuno-PET affording more specific whole-body images as compared to FDG-PET [12,13,14]. mAbs are promising vectors for theranostic approaches, to better identify patients who will respond to specific treatments and to monitor responses [13]. Based on immuno-PET, treatment strategies could be tailored for individual patients before administering expensive and potentially toxic therapies. Immuno-PET can offer a non-invasive solution to quantitatively assess target expression. Moreover, imaging plays an increasing role in the development of new drugs by pharmaceutical companies: *In vivo* imaging constitutes an effective solution for the rapid assessment of drug candidates, which may be radio-labeled to monitor their pharmacokinetics and biodistribution during preclinical and early clinical phases. The success of antibody-based radiopharmaceuticals for RIT or immuno-PET depends on progress in mAb production and targeting but also on the choice of the radionuclide. This review will thus describe some of these developments emphasizing the use of innovative radionuclides for tumor RIT and also presenting recent promising results of immuno-PET in theranostic approaches in the context of personalized medicine.

## 2. Principle of RIT

RIT is a molecular targeted radionuclide therapy whereby low dose rate-irradiation from radionuclides is delivered to tumor cells using mAbs directed to tumor antigens [15]. The cytotoxic mechanisms involve both radiobiological and immunological processes [16,17]. RIT delivers a heterogeneous low-dose-rate irradiation to the targeted tumor. Although a dose-effect relationship has not yet been clearly demonstrated, it is likely to be present even if such a relationship may be masked, in the treatment of B cell lymphoma, by the anti-tumor effects of cold mAbs generally injected prior to the radiolabeled antibody. Indeed, mAbs, particularly rituximab, may exert cytotoxic effects through apoptosis, antibody-dependent cell-mediated cytotoxicity and complement-dependent cytotoxicity. When mAbs are labeled with radionuclides, the combination of immunological and radiobiological cytotoxicity, including bystander and abscopal effects, results in higher anti-tumor efficacy [16].

Today, only two RIT-products targeting the CD20 antigen have been approved: the intact murine immunoglobulins ^131^I-tositumomab, (Bexxar^®^; GlaxoSmithKline, Mississauga, ON, USA) and ^90^Y-ibritumomab tiuxetan, (Zevalin^®^, Spectrum Pharmaceuticals, Henderson, NV, USA). Sales of ^131^I-tositumomab are now discontinued. ^90^Y-ibritumumab can be integrated in clinical practice using non-ablative activities for treatment of patients with relapsed or refractory follicular lymphoma (FL) or as consolidation after induction chemotherapy in front-line treatment in FL patients. Numerous studies showed also promising results in patients with FL and other aggressive B-NHL using high dose myeloablative regimens, RIT used as consolidation, RIT in first-line therapy or delivered using fractionation to increase cumulated injected activity and tumor absorbed dose without raising hematological toxicity [18]. Moreover, preclinical data and some pilot clinical studies suggested potential efficacy of RIT in other hemopathies, such as multiple myeloma (MM) or acute leukemia, especially using alpha emitters more adapted to target microscopic disease.

In solid tumors, more resistant to radiations and less accessible to large molecules such as mAbs, clinical efficacy remains limited. However, RIT used as consolidation therapy targeting minimal residual disease (MRD) achieved promising clinical efficacy in colon-rectum carcinoma (CRC) patients [19]. Prostate cancer (PCa) represents another favorable indication for RIT at this stage of MRD, occult disease being detectable by monitoring PSA serum level [20]. At the stage of MRD, activity uptake in tumors is faster and more favorable; tumor cells are less hypoxic and more radiosensitive. Pretargeting methods have also shown potential in CEA-positive tumors, such as medullary thyroid carcinoma (MTC) or CRC [15,21,22,23,24,25]. Fractionated protocols and combination with chemotherapeutic agents also demonstrated anti-tumor effects in patients with pancreatic adenocarcinoma [26].

## 3. Radionuclides and Labeling Techniques for RIT

### 3.1. Radionuclides

The efficiency of immunotargeting depends on several parameters, including the choice of the antigen target, the mAb (size, specificity, affinity, *etc.*) but the choice of an appropriate radionuclide also is critical [27,28]. Table 1 shows the different radionuclides used for RIT. As opposed to external beam radiotherapy, which uses penetrating radiations, such as photons, RIT uses short-range radiations from β- or α-particles. These particles deliver their energy within small distances, an ideal situation to preserve non-targeted tissues. The path-length of penetration of the radioactive emission should match the size of the targeted tumor. Today, only iodine-131 and yttrium-90 beta emitters received marketing authorization from regulatory authorities. Yttrium-90, with its long-range beta emission, is better suited for bulky disease. However, promising results have been observed using ^90^Y-RIT in the consolidation setting in patients in partial response (PR) or complete response (CR) after induction therapy [29,30]. Radionuclides such as ^177^Lu with shorter-range energy emissions should be more favorable in the setting of MRD. Moreover, ^177^Lu presents better physical properties than^131^I, improving the safety of RIT.

The rationale of alpha-RIT is based on two prominent characteristics of alpha particles: their short range in tissue, inferior to 100 μm, which allows for a good specificity of the treatment (once the antibody is in the vicinity of the tumor) and their high linear energy transfer (LET) between 50 and 250 keV/μm, which makes them highly cytotoxic [15,16,27,31,32]. In addition, alpha particle-induced toxicity was shown to be independent of both dose rate and oxygenation of the irradiated tissue [33]. The alpha-RIT appears relevant to target MRD or isolated tumor cells.

The half-life of radionuclide must also be considered. As, most often, radiopharmaceuticals are administered by systemic infusion, radioactive decay occurs along the course to the target, leading to non-specific irradiation of healthy tissues. This appeals for use of small carriers that quickly reach the target cells, as proposed in peptide receptor radionuclide therapy or pretargeting approaches [21]. By contrast, mAbs may take a couple of days for maximal uptake in target sites. Therefore, it is relevant to adjust the radionuclide physical half-life to the carrier biological half-life.

Taking all these criteria into account, very few radionuclides (Table 1) remain for RIT. Beta emitters such as ^131^I or ^90^Y have been used for a long time.^177^Lu and ^188^Re are emerging. ^67^Cu and a few other radionuclides are considered as very promising. Most are produced in nuclear reactors. When specific activity is of concern, indirect production routes can be used (for example ^177^Lu can be produced by neutron capture from ^176^Lu or by decay of ^177^Yb produced by neutron capture from ^176^Yb). ^188^W used as a precursor of ^188^Re needs very high neutron fluxes. Only ^67^Cu is produced in accelerators and is available part of the year from BNL (Upton, NY, USA) [34].

For alpha emitters, ^213^Bi is available through a generator made of ^225^Ac. Its short half-life makes it tricky to use, nonetheless many studies are ongoing worldwide [35]. Despite its complex chemistry, ^211^At may be a better candidate for alpha therapy due to its longer half-life and its production in accelerators. Other alpha emitters are available but they are linked to a cascade of alpha decays that may be a problem for specific targeting (^225^Ac and ^226^Th) or have chemical properties not favorable for labeling (^223^Ra).

**Table 1:** Radionuclides for antibody-targeted imaging and therapy.

Radionuclide	T_1/2_ (hours) ^a^	Main Emissions ^b^	E Max (keV)	Range Max in Soft Tissue (mm)	Usual Labeling Method
Fluorine-18	1.83	β^+^	633	3.1	*N*-hydroxy-succinimide ^18^F-fluoro-benzotate, click chemistry, ^18^F-aluminum-NOTA
Gallium-68	1.13	β^+^	1899	9.8	Polyamino-carboxylic acids: DOTA, NOTA
Copper-64	12.7	β^+^	653	3.2	Many different chelating agents
		β^−^	579	2.8	
Yttrium-86	14.7	β^+^	1220–2242	11	Polyamino-carboxylic acids: DOTA
Bromine-76	16.2	β^+^	1893 and 3382	19	Direct bromination, bromine-labeled activated esters
Zirconium-89	78	β^+^	902	4.6	Desferroxamine
Iodine-124	100	β^+^	1535 and 2138	7.9 and 10.9	Direct labeling (tyrosine)
Scandium-44	3.97	β^+^	1473	7.6	Polyamino-carboxylic acids: DOTA
Iodine-131	193	β^−^	610	2.9	Direct labeling (tyrosine)
		γ	362		
Yttrium-90	64	β^−^	2250	11	Polyamino-carboxylic acids: DOTA
Rhenium-188	17	β^−^	2120	10	Direct labeling or N2S2 or N3S complexes (chemistry analogous to that of technetium)
		γ	155		
Lutetium-177	162	β^−^	498	2.0	Polyamino-carboxylic acids: DOTA
		γ	208		
Copper-67	62	β^−^	392–577	1.8	Many different chelating agents
		γ	184		
Bismuth-212	1.01	α	6051 and 6090	0.07	Polyamino-carboxylic acids: CHX-DTPA, DOTA
		γ	727		
Bismuth-213	0.76	α	8,400	0.1	Polyamino-carboxylic acids: CHX-DTPA, DOTA
		γ	440		
Astatine-211	7.2	α	5870 and 7450	0.055–0.080	Stannylated synthons: SAB, SAPS
		X	77–92		
Actinium-225	240	α	+ alpha emitting daughters	*	Polyamino-carboxylic acids: DOTA
Thorium-227	449	α	+ alpha emitting daughters	*	Polyamino-carboxylic acids: DOTA
		γ			

* There is no clearly defined range for Actinium-225 and Thorium-227 because of multiple successive alpha emissions; ^a^ The half-life of the radionuclide must be matched with the half-life of its vector or more precisely, it should allow for clearance of unbound activity to obtain high target to non target tissue contrast ratio for imaging and it should be matched with the vector residence time in the tumor to deliver the maximum irradiation dose; ^b^ Intermediate energy photons (100–400 keV) may be detected by gamma cameras. Positron annihilation photon pairs may be detected by PET cameras. Only radionuclides emitting massive particles (alpha, beta, Auger electron) deliver their ionizing energy locally enough for therapy. In that case, concomitant emission of gamma or X rays may be used for imaging to check targeting and calculate irradiation doses absorbed by tumors and normal tissues.

Regarding the targeting aspect, the specificity of the radiopharmaceutical has to be the highest possible to limit the delivery of radionuclides to healthy tissues. On the other hand, affinity controls the uptake of the radiopharmaceutical in target lesions: higher affinity means higher uptake, although affinities in the nanomolar range are considered sufficient. For longer half-life radionuclides the rate of efflux from the tumor is also very important and is not entirely related to affinity. For instance, when the radiopharmaceutical is internalized by target cells, residualizing radionuclides, such as metals, afford protracted radioactivity retention in tumor sites, whereas direct radiolabeling with radioiodine result in fast excretion of radioactivity, thus reducing target cell exposure.

### 3.2. Labeling Techniques

Direct iodination by electrophilic substitution on a tyrosine residue is the easiest way to attach radioactive iodine to biological vectors [36]. Unfortunately, even if this commonly used technology is validated with non-internalizing antibodies or peptides, it does not provide satisfactory results when internalization occurs or with ^211^At-labeled antibodies [37]. In both cases, halogen liberation leads to non-specific irradiation of normal organs, such as thyroid for iodine or stomach for astatine, and reduces specific irradiation of the tumor. Several radiolabeling approaches using prosthetic groups have been proposed to solve this problem.

Radiometals, such as ^90^Y, ^188^Re, ^67^Cu, ^177^Lu, ^213^Bi and other actinides, are generally provided no-carrier added in chloride form or obtained from generators. However, contaminations with metal traces resulting from the production mode decrease the specific activity of radiopharmaceuticals, which are usually between 40 to 400 MBq/nmol depending on the radionuclide. Several highly specific chelating agents have been developed in order to improve specific activity [38].

Transmetallation or transchelation phenomena can occur *in vivo* when the radiopharmaceutrical is in competition with metal complexing proteins, such as transferrine or ceruleoplasmine. The best approaches to limit these phenomena are based on a better chelation agent selection in order to improve both selectivity and stability. This choice integrates dissociation constants and dissociation kinetics values, which have to be as low as possible. Thus chelating agents with very high affinities for metals and very high kinetic stabilities have been developed [38].

## 4. RIT Efficacy Using Innovative β^−^ Emitters

### 4.1. ^177^Lu-J591 Anti-PSMA in Metastatic Prostate Cancer (PCa)

PCa is a favorable solid malignancy for which RIT may be used because it is a radiosensitive tumor with typical distribution to sites with high exposure to circulating mAbs (bone marrow and lymph nodes). In preclinical and clinical PCa therapy studies, radionuclides have been linked to antibodies or peptides with affinity to mucin, ganglioside (L6), Lewis Y (Ley), adenocarcinoma-associated antigens, and Prostate Specific Membrane Antigen (PSMA) [20,39,40,41], but PSMA appears the most specific.

PSMA is an integral, non-secreted, type II membrane protein with abundant and nearly universal expression on prostate epithelial cells that is strongly upregulated in PCa [42,43,44,45,46]. Pathology studies indicate that PSMA is expressed by virtually all PCa [47]. The level of expression in non-prostate tissues is 100–1000-fold less than in prostate tissue, and the site of PSMA expression in normal cells (brush border/luminal location) are not typically exposed to circulating mAb. De-immunized J591 mAb, which targets the external domain of PSMA, seems to be the best clinical candidate for imaging and therapy of PCa [48,49].

Thirty-five patients were enrolled in a ^177^Lu-J591 phase I trial [50]. The 2590 MBq/m^2^ level was determined as maximal tolerated dose (MTD). Repeated dosing up to three doses of 1110 MBq/m^2^ could be safely administered. Clearly identified sites of metastatic disease were successfully imaged by ^177^Lu-J591 scintigraphy in 100% of patients. The median duration of PSA stabilization, after treatment, was 60 days with a range of 28 to 601 days. No immune response was detected. A phase II ^177^Lu-J591 trial has been performed in castration-resistant PCa patients [51]. Fifteen patients (cohort 1) were treated with 2405 MBq/m^2^. The second cohort (2590 MBq/m^2^) enrolled 17 patients expanded from 15 patients. Sensitivity of known metastasis targeting was 93.6%. Reversible thrombocytopenia and neutropenia toxicity occurred respectively in 46.8% and 25.5%. The second cohort dose (2590 MBq/m^2^) showed more PSA responses (46.9% *vs.* 13.3%, *p* = 0.048) associated with a longer survival (21.8 *vs.* 11.9 months, *p* = 0.03), but also more reversible hematologic toxicity. This trial supports that radiolabeled de-immunized J591 is well-tolerated and non-immunogenic. Radiolabeled J591 effectively targets PCa metastases with high sensitivity and specificity and produces PSA decline with a dose-effect relationship. 

### 4.2. Pretargeted ^177^Lu-Peptide in CEA-Positive Tumor

**Figure 1:**
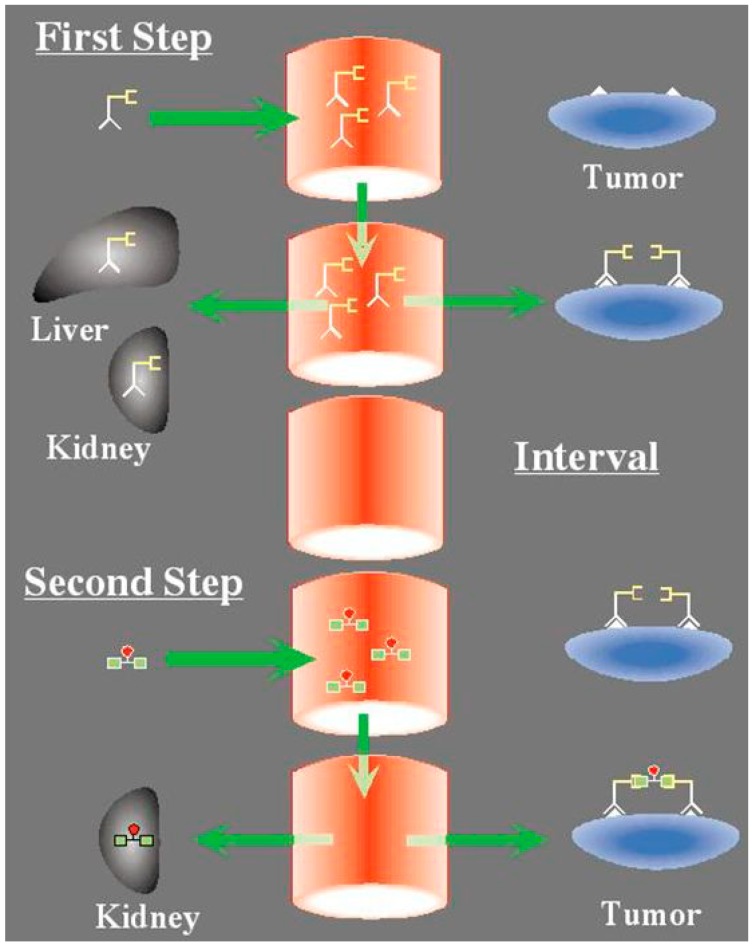
The concept of pretargeting with the Affinity Enhancement System: a bispecific antibody, designed to bind by one arm a tumor antigen (e.g., carcinomembryonic antigen) and by the other a hapten (e.g., the indium-diethylene triamine pentaacetic acid (DTPA) complex or the histamine-succinyl-glutamine (HSG) pseudo-peptide), is injected first. It distributes in the whole-body and targets the tumor cells. After an interval of several hours to a few days, the radiolabeled bivalent hapten is injected. It binds rapidly to the tumor. At the tumor cell surface, hapten bivalency induces cooperativity, resulting in very slow release.

Among several alternative possibilities, pretargeting may be achieved by a first injection of an unlabeled bispecific monoclonal antibody (BsMAb), followed by a second injection of a radiolabeled bivalent hapten-peptide [6,21]. Using this system, the radiolabeled bivalent peptide binds avidly to the BsMAb attached to the antigen at the cell surface, whereas non-targeted hapten-peptide in the circulation clears rapidly through the kidneys (Figure 1). MTC cells express high amounts of CEA and encouraging therapeutic results have been obtained using anti-CEA pretargeted ^131^I-di-DTPA peptide in 2 phase I/II and 1 phase II clinical trials [6,22,23,24].

Forty-five metastatic progressive MTC patients were enrolled in the prospective multicentric phase II assessing the chimeric hMN-14x734 BsMAb, followed by 1.8 GBq/m^2^ of ^131^I-di-DTPA given four to six days later [24,52]. According to RECIST morphological imaging criteria, 76.2% disease control rate (durable stabilization plus objective response) was obtained. One durable complete response of at least 40 months (2.4%) and 31 durable stable disease ≥6 months (73.8%) were observed in these patients with disease progression before RIT. Tumor uptake assessed by post-pretargeted RIT immunoscintigraphy predicted significantly the tumor response. After RIT, 21 of 37 assessed patients (56.7%) showed a ≥100% increase of doubling time biomarker serum concentration or prolonged decrease in biomarker serum concentration. As expected in these patients with a high frequency of diffuse bone marrow involvement, high-grade 3 and 4 hematologic toxicity was observed in 54.7% of patients, and myelodysplastic syndrome reported in two cases, including one heavily treated previously.

**Figure 2:**
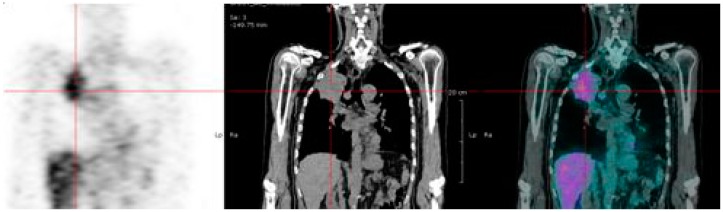
Images recorded in a patient with a carcino-embryonic antigen (CEA)-positive lung carcinoma treated by pretargeted radioimmunotherapy using the TF2 anti-CEA bispecific antibody and the ^177^Lu-IMP288 peptide. Image shows a good targeting of the lung tumor.

Today, new generation BsMAb and bivalent hapten-peptides are available. Humanized, recombinant, trivalent BsMAb and the histamine-succinyl-glutamine (HSG) hapten and bivalent HSG hapten-peptides have been produced [25,52,53,54,55,56,57]. The use of humanized BsMAb should reduce immunogenicity and the Dock-and-Lock procedure allows large-scale production. Then, a series of bivalent HSG haptens have been synthesized, offering the possibility of labeling with a variety of radionuclides, including yttrium-90 and lutetium-177 for therapy purposes [56]. The first clinical results of an optimization study assessing the anti-CEA × anti-HSG bsMAb TF2 and the radiolabeled hapten-peptide, ^177^Lu-IMP288, in patients with metastatic CRC have been reported recently [25,57]. Different schedules were studied in four cohorts of five patients, to define the optimal molar doses of TF2 and IMP-288 and the optimal delay between the two infusions: (1) shortening the interval between the bsMAb and peptide administration (one to five days); (2) escalating the TF2 dose (from 75 to 150 mg); and (3) reducing the peptide dose (from 100 to 25 μg). Rapid and selective tumor uptake was detected within 1 h after the peptide injection, with high tumor-to-tissue ratios at 24 h. The best tumor targeting was achieved with a one-day pretargeting interval and with the 25-μg peptide dose. High activities of ^177^Lu-IMP288 (2.5–7.4 GBq) were well tolerated, with some manageable reactions during the TF2 infusions, and transient grades 3–4 thrombocytopenia in 10% of the patients. Dosimetry analysis concluded that renal and red bone marrow uptake of ^177^Lu-IMP288 peptide was relatively low, even if marrow doses increased in subsequent cohorts as the TF2/Lu-IMP288 ratio was increased [57]. The predicted kidney absorbed doses (<0.50 mGy/MBq) did not limit the maximum activity that could be administered. None of the patients would exceed the limit of 15 Gy to the kidneys with four cycles of 7.4 GBq ^177^Lu-IMP288. These data suggest that pretargeting using Dock-and-Lock bispecific BsMAb would be most useful to deliver short half-life radionuclides. In preclinical studies with lutetium-177, the limited specific activity was overcome by using repeated injections of both the bispecific antibody and IMP 288 labeled with lutetium-177 [56]. Also, yttrium-90 could perform better than lutetium-177 in this context and short half-life alpha-particle-emitting radionuclides, such as astatine-211 or bismuth-213, should be considered [58]. In France, two multicentric prospective phase-I clinical studies are on-going, assessing pretargeted ^177^Lu-IMP288 (one injection) in patients with metastatic CEA-positive lung carcinoma and fractionated injection of ^90^Y-IMP288 in metastatic CRC patients. Figure 2 shows images recorded in a patient with a lung carcinoma included in the on-going clinical trial assessing pretargeting ^177^Lu-IMP288.

### 4.3. Interest of ^67^Cu for RIT

Copper-67 has favorable radiophysical characteristics for RIT with a half-life of 3.4 days well adapted to pharmacokinetics of whole antibodies and an emission of beta particles with energy comparable to that of iodine-131 and lutetium-177. Moreover it emits gamma rays with energy suitable for imaging and a relatively weak abundance thus avoiding irradiation of patients and medical staff like with iodine-131. Production capacity of copper-67 is limited by a low cross section of the nuclear reaction, which needs high intensity and high energy proton beams. Only a limited number of accelerators in the world have such high energy and high intensity. This situation explains the quite limited number of clinical studies performed in the last 30 years.

Two groups of investigators carried out clinical studies in the 1990s in a limited number of patients. De Nardo and coworkers were the pioneers and compared clinical results obtained with Lym-1 directed to NHL and labeled with Cu-67, I-131 and Y-90 [59,60]. The authors concluded that the therapeutic indices (ratio of radiation doses to tumor and normal tissues) for ^67^Cu-2IT-BAT-Lym-1 and less generally for ^90^Y-2IT-BAD-Lym-1 were more favorable when compared to those for ^131^I-Lym-1. The same conclusions were drawn in a clinical study comparing the anti-CEA mAb35 antibody labeled with Cu-67 and I-125 in patients with CRC [61]. Copper-67-labeled mAb35 was more favorable than its radioiodine-labeled counterpart due to higher tumor-to-blood ratio but the authors observed a potential problem with Cu-67 due to non-specific liver and bowel uptake.

So copper-67 seems more suitable than iodine-131 and possibly then yttrium-90 for the labeling of MAbs, but currently lutetium-177 is a radionuclide of choice for RIT. Thus in the future, copper-67 should be compared to lutetium-177 in clinical studies when large activities of copper-67 will be produced by new high-energy/high-intensity accelerators, such as the Arronax cyclotron in Nantes, France.

### 4.4. Other Radionuclides

A few other radionuclides could be of interest for RIT. For example, scandium-47 has been proposed by Pietrelli *et al.* [62]. It may be produced carrier free by neutron irradiation of titanium-47. It decays into stable titanium by emitting low energy β^−^ with a half-life of 3.35 days and a γ-ray at 159 keV (68%) that is suitable for imaging. Very little has been published on scandium-47 because of a poor availability, but interest in scandium-44, a positron emitter, has revived interest in scandium-47 according to the β^+^/β^−^ radionuclide pair concept. Like scandium-44, it may be efficiently complexed by several chelating agent, including DOTA [63].

Terbium-161 has also been proposed recently as a β^−^ emitter of interest in targeted radionuclide therapy [64]. It decays with a half-life of 6.88 days into stable dysprosium-161 by emitting low energy β^−^. A very interesting feature of terbium-161 is the possibility of using other terbium radionuclides, such as terbium-149, which is an alpha emitter, terbium-152 a positron emitter, and terbium-155 a gamma emitter, all with manageable half-life and decay properties, thus making it possible to perform β^−^ and alpha targeted radionuclide as well as PET and SPECT with isotopes of the same element.

## 5. RIT with Alpha-Emitting Radionuclides

Related to these characteristics, it is often described that alpha-RIT is particularly indicated in the treatment of MRD, hematologic cancers and micrometastatic diseases, even though some efficacy was observed on solid tumors. Although medical applications of alpha emitting radionuclides in medicine had been contemplated in the early 20th century, just after the discovery of radioactivity, the first alpha-RIT clinical trial was performed in 1997. A humanized antibody specific for a human myelogenous leukemia antigen (CD33) labeled with ^213^Bi was administered to 18 patients with Acute Myeloid Leukemia and results showed a reduction in circulating blasts in most patients (~80%), whereas no extramedullary toxicity was observed [65]. Recently, efficacy of ^213^Bi-RIT was observed in a syngeneic multiple myeloma mouse model using an anti-mouse CD138 MAb labeled with ^213^Bi-RIT [66,67]. ^213^Bi-RIT administered 10 days after engraftment increased survival, with only moderate and transient hematological toxicity. Similarly, a clinical study assessed ^213^Bi-DOTA-substance P injected in the tumor via implanted catheters in five patients with WHO grade II-IV gliomas, showing radiation-induced tumor necrosis [67].

Actinium-225 is also considered as an interesting alpha emitter. McDevitt *et al.* proposed the concept of an *in vivo* nanogenerator, using a mAb derivatized with DOTA, that demonstrated the retention of actinium-225 and its radioactive daughters in the targeted tumor cell after internalization [68,69]. Two clinical studies are ongoing in the USA with an anti-CD33 antibody labeled with actinium-225 in patients with Acute Myeloid Leukemia. Alpha-RIT using Lead-212/Bismuth-212 generator is also assessed, in an on-going phase I trial, using anti-HER2 radio-labeled Mab intra-peritoneally injected in patients with HER-2-expressing peritoneal carcinomatosis for which no standard therapy is available [70,71]. A potential problem of ^225^Ac is its short-lived daughter ^221^Fr. This nuclide would be rapidly in equilibrium with the mother nuclide and could be re-distributed in the body. Contribution of errand daughters (bismuth-213) to kidney dosimetry has already been mentioned [72]. Thorium-227 is another *in vivo* generator that may solve the problem because its major alpha-emitting daughter is radium-223 a long lived radionuclide, with proven good tolerance [73]. A recent preclinical study reported the contribution of Mab internalization in the intra-peritoneal ^212^Pb-RIT efficacy of small volume carcinomatosis, comparing targeting of HER2 (internalizing) or CEA (non-internalizing) [28,74]. An advantage was observed using internalizing anti-HER2 compared with non-internalizing anti-CEA ^212^Pb-labeled antibodies.

Astatine-211, an alpha emitting radionuclide with a physical half-live of 7.2 h, also appears relevant for RIT. In 1989, labeling of a mAb with ^211^At was reported using *N*-succinimidyl 3-(trimethylstannyl)benzoate as a synthon [75]. The same research group labeled an internalizing mAb with ^211^At using *N*-succinimidyl 5-[^211^At]astato-3-pyridinecarboxylate ([^211^At]SAPC) as a synthon, showing that astatine-211 was intracellularly retained after internalization [76]. Recently, preclinical studies showed that anti-CD45 ^211^At-RIT and bone marrow transplantation prolonged survival in a disseminated Acute Myeloid Leukemia murine model [77]. Biodistribution studies showed excellent localization of the ^211^At-anti-murine CD45 mAb 30F11 to marrow and spleen within 24 h. In syngeneic hematopoietic stem cell transplantation studies, ^211^At-RIT improved the median survival of leukemic mice in a dose-dependent fashion, with minimal toxicity. ^211^At-RIT feasibility was reported in two clinical trials. The first study assessed anti-tenascin ^211^At-RIT followed by chemotherapy in patients with glioblastoma [78]. The radioimmunoconjugate was injected into the resection cavity. The maximal injected activity was 347 MBq (9.4 mCi). Six patients out of 18 had a reversible grade 2 neurotoxicity but no grade 3-4 toxicities were observed. Maximal tolerated activity was not reached and observed median survival favorably compared with that of historical control groups. In the second study, ^211^At-MX35 F(ab')_2_ was assessed in women in CR after a second-line chemotherapy for recurrent ovarian carcinoma in a phase I study [79]. The aim was to determine dosimetry and toxicity. ^211^At was labeled to MX35 F(ab')_2_ using the reagent *N*-succinimidyl-3-(trimethylstannyl)-benzoate. Nine patients underwent laparoscopy two to five days before ^211^At-RIT. Before the RIT infusion, the abdominal cavity was inspected to exclude the presence of macroscopic tumor growth or major adhesions. Patients were infused with ^211^At-MX35 (22.4–101 MBq/L) in dialysis solution via the peritoneal catheter. The estimated absorbed dose to the peritoneum was 15.6 ± 1.0 mGy/MBq/L), to red bone marrow 0.14 ± 0.04 mGy/MBq/L) and to the unblocked thyroid 24.7 ± 11.1 mGy/MBq/L). This dose decreased when the thyroid was blocked (1.4 ± 1.6 mGy/MBq/L). No adverse effects were reported. This study indicates that intraperitoneal ^211^At-RIT delivers therapeutic absorbed doses in microscopic tumor clusters without significant toxicity. A third phase I clinical study is in preparation in Nantes, France, aiming at evaluating anti-PSMA ^211^At-J591 in patients with progressing metastatic PCa.

## 6. Immuno-PET for Tumor Imaging and Theranostic Approaches

### 6.1. Interest of Immuno-PET and Choice of Radionuclides

For more than two decades, mAbs have been labeled with gamma-emitting radionuclides, such as ^131^I or ^111^In, and subsequently used in planar or Single Photon Emission Computed Tomography (SPECT) imaging procedures. While providing reliable information, these imaging modalities suffer from poor sensitivity and poor spatial resolution. Accurate quantitative information could be better obtained using PET. The improved spatial resolution makes the delineation of tumors and organs better, as compared to SPECT [80,81,82,83]. Additionally, exact attenuation correction, precise scatter correction and, last but not least, high sensitivity combined with the possibility to perform true whole body imaging in a reasonable time constitute the key factors for the superiority of PET over SPECT. PET imaging also takes advantage of new advances in PET detectors [80,81] and reconstruction algorithm that improve spatial resolution and signal-to-noise ratios. However combining mAbs and PET emitters for immuno-PET requires an appropriate match between the biologic half-life of the vector and the physical half-life of the isotope [13,83,84]. Table 1 shows different relevant PET emitters. The use of ^18^F or ^68^Ga with a short half-life is limited to small size molecules such as peptides or small molecular weight binding proteins that distribute rapidly in the body [54,55,85,86,87], whereas ^89^Zr [88,89] and ^124^I [90,91,92] are well suited to the labeling of large molecules, such as intact mAbs. Copper-64 with an intermediate half- life of 12.7 h can be used for labeling of a large number of molecules with different sizes [93]. Within the scope of a “theranostic” approach, pairs of beta+/beta− emitting radionuclides (^124^I/^131^I, ^86^Y/^90^Y, ^64^Cu/^67^Cu, ^44^Sc/^47^Sc) are very promising because the same distribution is expected both for imaging dosimetry and therapy with the same elements. Immuno-PET can offer a non-invasive solution to quantitatively assess target expression. It is also a powerful innovation for improving knowledge about efficacy and *in vivo* behavior of mAbs. Based on immuno-PET, the treatment strategy could be tailored for individual patients before administering expensive medicines. Immuno-PET also represents a non-invasive technique for monitoring tumor response by measuring early changes in biomarker expression before being detected using MRI or CT [94]. Immuno-PET also represents a relevant tool when multi-observation image analysis is considered. This emerging field aims at merging several PET acquisitions to assess tumor characterization (as metabolic volume, uptake variations or heterogeneity).

### 6.2. ^68^Ga-Peptide for Pretargeted Immuno-PET in CEA Positive Tumors

**Figure 3:**
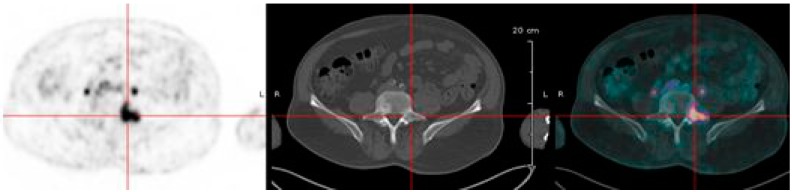
Positron emission tomography (PET) in a patient with a relapse of medullary thyroid carcinoma recorded after injection of the TF2 anti-carcino-embryonic antigen (CEA) bispecific antibody and the ^68^Ga-IMP-288 peptide. Image shows a good detection of a bone lesion.

**Figure 4:**
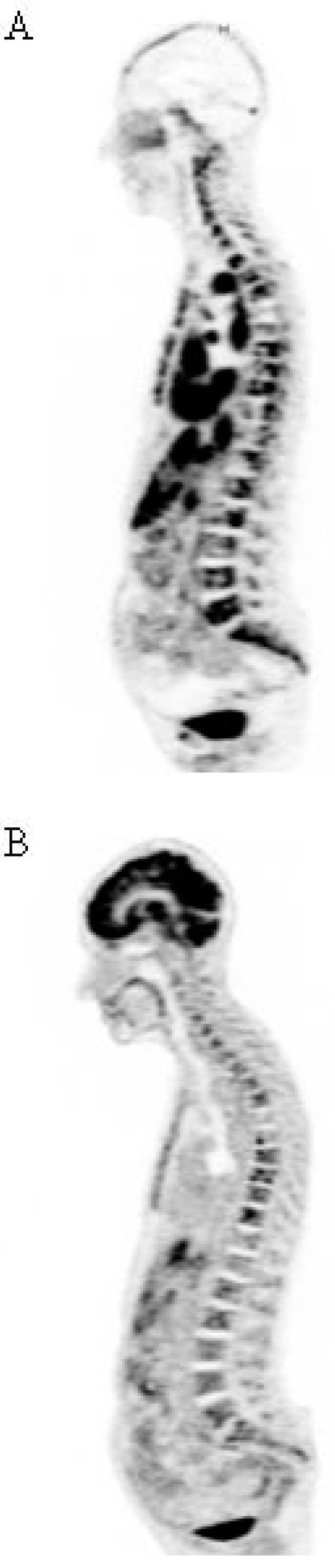
Imaging performed in a patient with a metastatic breast carcinoma. (**A**) Immuno-PET performed using the TF2 anti-CEA bispecific antibody and the ^68^Ga-IMP-288 peptide detects a more diffuse bone marrow involvement that FDG-PET (**B**).

Pretargeted immuno-PET can be performed with the HSG peptides previously presented labeled with short-lived PET emitters, such as ^18^F or ^68^Ga, as shown in preclinical models [55,87]. Pretargeting needs a clinical optimization of reagent molar doses and pretargeting delay. Two clinical trials are on-going in France, aiming at optimizing ^68^Ga-IMP288 and the anti-CEA trivalent humanized TF2 BsMAb molar doses and pretargeting delay for immuno-PET in patients with relapsed MTC or HER2-negative BC. Preliminary results suggested that a 30-h pretargeting delay was optimal [95]. In MTC, immuno-PET detected tumor lesions not detected by F-DOPA-PET, considered as a reference PET imaging radiopharmaceutical in MTC (Figure 3). Promising results were also obtained in CEA-positive breast cancer patients (Figure 4). Performance of pretargeted immuno-PET compared also favorably with conventional Imaging by computed tomography or MRI. Another clinical study will start in France in 2015, assessing pretargeted immuno-PET sensitivity in metastatic CRC.

### 6.3. Immuno-PET of Carbonic Anhydrase IX for Renal Masses Exploration

The chimeric antibody cG250 (girentuximab) binds carbonic anhydrase IX, a cell-surface antigen highly and homogeneously expressed in more than 95% of clear cell renal cell carcinoma (ccRCC). Immuno-PET using ^124^I-girentuximab demonstrated, in 26 presurgical patients with renal masses, a sensitivity of 94% and a specificity of 100%, with a negative predictive value of 90% and a positive predictive value of 100% [91]. On the basis of these data, The REDECT phase III multicenter trial has been conducted, assessing ^124^I-girentuximab immuno-PET in a contemporary cohort of patients with renal cortical tumors. ^124^I-girentuximab was well tolerated. The data were available for a complete analysis in 195 patients. For immuno-PET, the sensitivity and the specificity were 86.2% and 85.9%, respectively, and for contrast-enhanced CT 75.5% and 46.8%, respectively (*p* = 0.23 and *p* = 0.05, for sensitivity and specificity, respectively). Interestingly, immuno-PET uses a qualitative dichotomous classification, facilitating the interpretation. Indeed, ^124^I-girentuximab immuno-PET provides information on the presence or absence of ccRCC with accuracy at least comparable to that of biopsy, allowing avoiding this invasive procedure with its inherent risks.

A recent publication suggested the interest of the radiometal ^89^Zr to label cG250 mAb, because of the internalization mechanism. Experimentation comparing ^89^Zr-cG250 and ^124^I-cG250 were conducted in mice bearing subcutaneous human ccRCC tumors [94]. The two tracers demonstrated virtually identical tumor cell binding and internalization but showed markedly different retentions *in vitro*. Superior immuno-PET images were obtained using ^89^Zr, because of the longer tumor trapping of the radiometal and simultaneous washout from normal tissues.

Because carbonic anhydrase IX expression in most other tumor types is generally correlated with hypoxia, the capability of ^89^Zr-cG250 to visualize tumor hypoxia has also been assessed, in head and neck tumor animal model [96]. In this preclinical study, the F(ab')_2_ fragment of cG250 mAb was used, and as early as 4 h after injection, tumor accumulation of ^89^Zr-cG250 was obtained. A significant positive correlation between ^89^Zr-cG250 distribution and carbonic anhydrase IX expression was observed (*r* = 0.57–0.74; *p* < 0.0001) and a less strong correlation with pimonidazole staining that reflects hypoxia (*r* = 0.46–0.68; *p* < 0.0001). Thus cG250 immuno-PET could be considered as a potential tool in defining carbonic anhydrase IX-positive hypoxic areas requiring intensified therapy and redistribution of radiation dose.

### 6.4. Companion Anti-HER2 PET in Breast Cancer (BC)

Immuno-PET could also be considered to predict response and select patients before targeted MAb therapy [95]. Targeting of HER2 illustrates this approach, anti-HER2 therapeutic agents being only effective in patients who have Her2-positive BC. Although HER2 expression is determined in clinical practice using immunohistochemistry, technical problems can arise when lesions are not accessible to biopsy and HER2 expression can vary during the disease history and across lesions within the same patient. It has been proven that immuno-PET with ^68^Ga, ^64^Cu or ^89^Zr could non-invasively identify lesions that are likely to respond to therapy [86,93,97]. The feasibility of anti-HER2 ^64^Cu-DOTA-trastuzumab was assessed in six patients with primary or metastatic HER2-positive BC [93]. Around 130 MBq was injected. No drug-related adverse events were reported. Radiation exposure during ^64^Cu-DOTA-trastuzumab immuno-PET was equivalent to that during FDG PET. Biodistribution in liver, spleen, and kidney was as expected. Interestingly, liver uptake was higher in the two patients not previously treated with trastuzumab. Tumor uptake showed better contrast at 48 h after the injection than at 24 h. In two patients, immuno-PET detected brain metastases, indicative of blood-brain barrier disruptions. Because of the shorter half-life of ^64^Cu as compared to ^89^Zr and the absence of high energy gamma emission, ^64^Cu-DOTA-trastuzumab appears to be more acceptable in clinical practice as compared with ^89^Zr-trastuzumab in terms of radiation exposure injection, estimated at 12 mSv with ^64^Cu-DOTA-trastuzumab and 18 mSv with ^89^Zr-trastuzumab PET [97,98]. However, the shorter half-life of ^64^Cu provides images with high activity in the blood, reducing the image contrast, but pre-dosing with unlabeled trastuzumab significantly decreases background in liver [98].

## 7. Conclusions

Radio-labeled immunoconjugates know several developments in oncology, because of improvement in mAb technology, but also in access to innovative radionuclides for therapy and imaging applications. The use of alternative beta emitters, such as ^177^Lu, ^67^Cu, or alpha-emitters, such as ^211^At, may improve RIT efficacy for treatment of hemopathies and solid tumors, especially at the stage of minimal disease. RIT may have the potential of killing the last tumor cells, now identified as chemoresistant and radioresistant tumor stem cells. Immuno-PET could probably help selecting patients for RIT and optimizing injected activities. mAbs labeled with PET emitters could also be used for *in vivo* molecular diagnosis or as companion in theranostic approaches, in the context of targeted Mab therapies, to assess non-invasively *in vivo* tumor antigen expression and accessibility to mAbs.
